# Phytochemicals from Red Onion, Grown with Eco-Sustainable Fertilizers, Protect Mammalian Cells from Oxidative Stress, Increasing Their Viability

**DOI:** 10.3390/molecules27196365

**Published:** 2022-09-27

**Authors:** Maria Laura Matrella, Alessio Valletti, Federica Marra, Carmelo Mallamaci, Tiziana Cocco, Adele Muscolo

**Affiliations:** 1 Department of Basic Medical Sciences, Neurosciences and Sense Organs, Biochemistry Section, University of Bari “Aldo Moro”, 70124 Bari, Italy; 2 Department of AGRARIA, Mediterranea University, Feo di Vito, 89122 Reggio Calabria, Italy

**Keywords:** red onion, phytochemicals, polyphenols, oxidative stress, H9c2 rat cardiomyoblast, primary human fibroblasts

## Abstract

Red onion, a species of great economic importance rich in phytochemicals (bioactive compounds) known for its medicinal properties, was fertilized with sulphur-bentonite enriched with orange residue or olive pomace, with the aim of producing onion enriched in health beneficial compounds. There is a worldwide great demand of minimally processed food or food ingredients with functional properties because of a new awareness of how important healthy functional nutrition is in life. Phytochemicals have the capacity to regulate most of the metabolic processes resulting in health benefits. Red onion bioactive compound quantity and quality can vary according to cultivation practices. The main aims of the current research were to determine the chemical characteristics of the crude extracts from red onion bulbs differently fertilized and to evaluate their biological activity in normal and oxidative stress conditions. The lyophilized onion bulbs have been tested in vitro on two cellular models, i.e., the H9c2 rat cardiomyoblast cell line and primary human dermal fibroblasts, in terms of viability and oxygen radical homeostasis. The results evidenced different phytochemical compositions and antioxidant activities of the extracts obtained from red onions differently fertilized. Sulphur-bentonite fertilizers containing orange waste and olive pomace positively affected the red onion quality with respect to the red onion control, evidencing that sulphur-bentonite-organic fertilization was able to stimulate plant a secondary metabolism inducing the production of phytochemicals with healthy functions. A positive effect of the extracts from red onions treated with fertilizers—in particular, with those containing orange waste, such as the reduction of oxidative stress and induction of cell viability of H9c2 and human fibroblasts—was observed, showing a concentration- and time-dependent profile. The results evidenced that the positive effects were related to the phenols and, in particular, to chlorogenic and p-coumaric acids and to the flavonol kaempferol, which were more present in red onion treated with low orange residue than in the other treated ones.

## 1. Introduction

Nowadays, there is an increasing attention on the food we eat. There is a worldwide great demand of minimally processed food or food ingredients with functional properties because of a new awareness of how a healthy and sustainable living is important. Bioactive food compounds, also known as phytochemicals, have the capacity to regulate most of the metabolic processes resulting in health benefits. So far, about 10,000 phytochemicals have been identified, but a large percentage remains still unknown. The identified phytochemicals include tannins, flavones, triterpenoids, steroids, saponins, and alkaloids. Numerous studies have associated the protective and beneficial roles of phytochemicals with their antioxidant activity, since the overproduction of oxidants (reactive oxygen species and reactive nitrogen species) in the human body is the cause of cellular aging [1], and of many chronic diseases [2]. Antioxidant phytochemicals exist widely in fruits, vegetables, cereal grains, edible macrofungi, microalgae, and medicinal plants. Among the vegetables rich in bioactive compounds, *Allium cepa* L. (the common onion) is one of the oldest plants cultivated around the world and consumed as a vegetable and spice. It is greatly appreciated as a medicinal plant in traditional medicine for its high content of phytochemicals, including polyphenols, flavonoids, and sulphur-based compounds. These secondary metabolites, widely contained in onions, have a different mode of action and biosynthetic pathways but are all able to promote beneficial health effects. A regular onion bulb intake is reported to have profound radical scavenging activity and several beneficial effects on health [3], such as preventing cardiovascular diseases [4], diabetes [5], cancers [6], and neurodegeneration [7]. Therefore, foods in our diet that can aid in the prevention of these diseases are of major interest to both the scientific and public communities.

Epidemiological data evidenced that a high intake of onions was positively correlated with a low risk of carcinoma [8,9]. Hertog and Katan [10] showed that a high consumption of quercetin-rich onion was associated with a 50% cancer risk reduction of the digestive and respiratory tracts. Organosulphur compounds such as diallyl disulfide (DDS), S-allylcysteine (SAC), and S-methylcysteine (SMC) have been demonstrated to inhibit colon and renal carcinogenesis [11,12]. Phytochemicals act through two different mechanisms: cancer cell apoptosis induction [13] and gene transcription inhibition [14].

The quantity and quality of bioactive compounds contained in the onion bulb can vary according to the variety and cultivation practices. Among the varieties, it was well-demonstrated that *A. cepa L. var. tropeana* (red onion) contains more phytochemicals than white onion [15]. With respect to the cultivation conditions, numerous researchers have evidenced that onion is a sulphur-loving crop and that sulphur increased the bulb yield quality and flavors. Other works indicated an increase in onion quality when organic fertilizers were used [16]. Muscolo et al. [17] showed that the use of sulphur-organic-based fertilizers increased, in red onion, the production of bioactive organosulphur compounds and antioxidants with respect to the type and concentration of sulphur-organic-based fertilizer used.

Based on the above findings, the main aims of the current research were to: (1) determine the chemical characteristics, phytochemical amount, and profile of red onion bulbs differently fertilized and (2) evaluate their biological activity in normal and oxidative stress conditions. The lyophilized onion bulbs were tested in two cellular models, i.e., the H9c2 rat cardiomyoblast cell line and primary human control, and *parkin*-mutant fibroblasts in terms of viability and oxygen radical homeostasis. H9c2 cells are a valid alternative for primary cardiomyocytes where oxidative stress is an important pathophysiological pathway, affecting multiple aspects of cardiac functionality, including signal transduction, cell cycle arrest, apoptosis, and necrosis [18,19]. In Parkinson’s disease (PD), oxidative stress plays a significant role in the cascade, consequently leading to the degeneration of dopaminergic neurons. Moreover, other aspects of the degenerative process, such as mitochondrial malfunction, excitotoxicity, nitric oxide toxicity, and inflammation, are all linked to oxidative stress [20]. Our goal was to link the protective benefits of red onion to the phytochemical content and specific class of compounds in order to emphasize the medicinal worth of these onions, which may be used in a health prevention program.

## 2. Results and Discussion

### 2.1. Red Onion Chemical Properties

The treatments with fertilizer pads, SB, SBOR and SBOP, influenced positively, but to different extents, the properties of red onions compared to the control (CTR). Pads containing orange positively affected the red onion quality, followed by SBOP and SB. These were due to the presence of organic components in the pads, as reported in previous publications [21,22] that evidenced a great level of flavonoids in organically grown Welsh onions and red onion. Ren et al. [21] also found high amounts of phenolics, total flavonoids, and anthocyanins, as well antioxidant activities, in two different onion varieties grown under organic production. Muscolo et al. [17] evidenced a positive effect of sulphur bentonite-organic-based fertilizers on secondary metabolite (SMs) production in red onions, suggesting that sulphur bentonite organic fertilization was able to stimulate the plant’s secondary metabolism, inducing the production of phytochemicals that can be useful in preserving human health. Human natural antioxidant systems, if perfectly working, are able to mitigate damage to important biomolecules, such as DNA, proteins, lipids, and carbohydrates, avoiding the insurgence of diseases [23]. The additional intake of antioxidants with the diet represents a very important way to prevent the diseases caused by oxidative stresses. There is, nowadays, a growing interest to enrich the human diet with functional foods naturally rich in antioxidant compounds. Polyphenols represent the most important natural antioxidant compounds with beneficial effects on human health [24].

**Figure 1:**
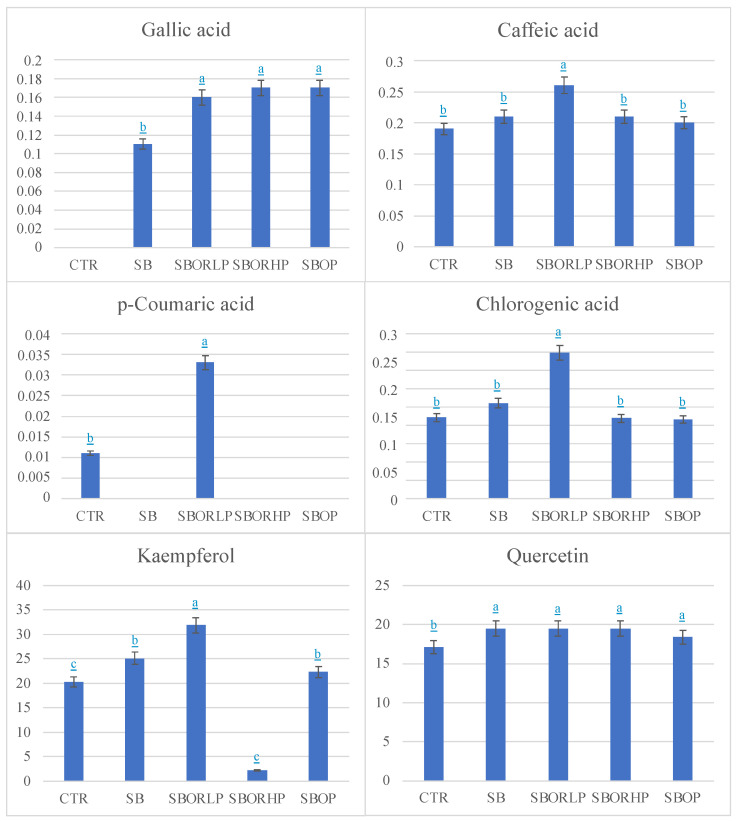
Phenolic acids and flavonols (mg∙100∙g^−1^ FW) found in red onion bulbs differently fertilized: control (CTR), sulphur bentonite (SB), sulphur bentonite-low percentage orange residue (SBOR LP), sulphur bentonite-high percentage orange residue (SBOR HP), and sulphur bentonite—olive pomace (SBOP). Data are the mean of three replicates ± the standard error. Different letters indicate significant differences at *p* < 0.05.

Our results evidenced in red onion bulb the greatest increase in polyphenols (Table 1) in the presence of SBOR at both concentrations (low and high); SBOP also increased the quantity of polyphenols with respect to the control but less than SBOR. In contrast, an inverse trend was observed for the total flavonoids (Table 1) that increased more in the presence of SBOP than SBOR LP and HP. Anthocyanins were the highest in all fertilized red onion bulbs. Phenolic acids (Figure 1) found in the CTR and fertilized onions were caffeic and chlorogenic. Gallic acid was present only in the fertilized onions (Figure 1), while *p*-coumaric acid in the CTR and, in the greatest amount, in SBORLP. Caffeic and chlorogenic acids did not show significant differences with respect to the CTR, except for the onion fertilized with SBORHP. *p*-coumaric and gallic acids are antioxidants with diverse physiological functions that are beneficial for human health with ascertained anticancer, anti-inflammatory, and antimicrobial properties [25,26,27]. The mechanisms of action of polyphenols are various and complex and depend on their chemical structures. The antioxidant property of *p*-coumaric acid is ascribed to its phenyl hydroxyl group (-OH) that enables it to donate hydrogen or electrons. In vivo studies on the *p*-coumaric mechanism of action evidenced, on a rat model, that it was able to reduce basal oxidative DNA damage, inducing glutathione (GSH) and glutathione S-transferase Mu 2 (GST-M2) in colonic mucosa. Additionally, it was demonstrated that *p*-coumaric acid was capable of decreasing the expression of the inflammatory mediators, such as TNF-α and IL-6, regulating the production of cytokines [28]. Nasr Bouzaiene et al. [29] showed how the proliferation of human lung (A549) and colon (HT29-D4) cancer cells was significantly inhibited by ferulic, caffeic, and *p*-coumaric acids. These inhibitory effects were likely to be mediated by the suppression of DNA synthesis induced by the phenolic acids in MCF-7. Caffeic acid, among the phenolic acids, was found more able to block the many modulators involved in tumor progression, including NF-kB, COX-2, TNF-a, IL-6, Nrf2, iNOS, NFAT and HIF-1α, repressing cancer angiogenesis and therefore recognized as an inducer of tumor cell death and performer of cancer growth blockage [30].

**Figure 2:**
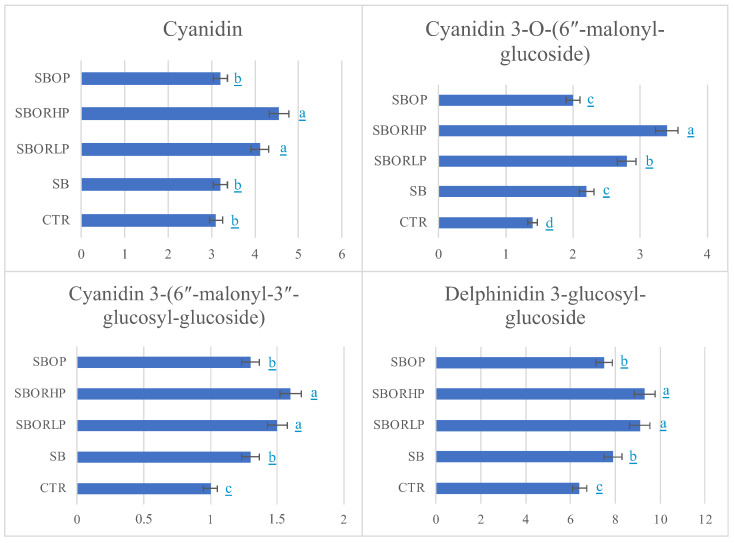
Anthocyanidins (mg∙100∙g^−1^ FW) found in red onion bulbs differently fertilized: control (CTR), sulphur bentonite (SB), sulphur bentonite-low percentage orange residue (SBOR LP), sulphur bentonite-high percentage orange residue (SBOR HP), and sulphur bentonite-olive pomace (SBOP). Data are the mean of three replicates ± the standard error. Different letters indicate significant differences at *p* < 0.05.

Anthocyanidins (Figure 2), increased in treated onions compared to the CTR. Equally, S methyl-cysteine sulfoxide and the majority of organosulphides (Table 2) increased with respect to the CTR, mostly in red onions treated with SBOR pads and particularly with SBORLP. Anthocyanidins have health-promoting effects linked with antioxidant, anti-inflammatory, and anticarcinogenic properties. Their antioxidant nature was observed in all neurological diseases through MMP2, MMP3 and MMP9 metalloproteinase inhibition; reactive oxygen species generation inhibition; endogenous antioxidants modulation as superoxide dismutase and glutathione; the formation and aggregation of beta-amyloid (β-A) protein inhibition; and brain protective action through the modulation of brain-derived neurotrophic factor (BDNF), important for neural plasticity [31]. Additionally, organosulphur compounds have a well-recognized antiproliferative activity in several tumor cell lines that is mediated by the induction of apoptosis and alterations of the cell cycle. Organosulphur compounds generally act by modulating the activity of several metabolizing enzymes that activate (cytochrome P450s) or detoxify (glutathione S-transferases) carcinogens and inhibit the formation of DNA adducts in several target tissues [32]. Their low amounts found in SBORHP and SBOP treated onions can be related to the contemporary increase in other SMs with antioxidant properties. This suggests that the fertilizers used were able to influence the biosynthesis and accumulation of other SMs, evidencing that these fertilizers are capable of redirecting the metabolism to consequently regulate the production of specific bioactive constituents, as already reported by [33].

**Figure 3:**
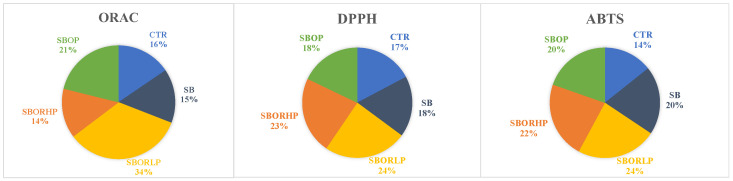
Antioxidant activities (ORAC, DPPH and ABTS) detected in red onion bulbs differently fertilized: control (CTR), sulphur bentonite (SB), sulphur bentonite-low percentage orange residue (SBOR LP), sulphur bentonite-high percentage orange residue (SBOR HP), and sulphur bentonite-olive pomace (SBOP). Data are the mean of three replicates.

The in vitro antioxidant capacity, determined with DPPH, ABTS and ORAC (Figure 3), increased in red onion grown mainly with SBOR and SBOP than the CTR (Figure 3). Specifically, ORAC was the highest in bulbs of red onion grown with SBOR LP, while DPPH and ABTS were the highest in bulbs of red onion grown with SBOR, both LP and HP. Cavalheiro et al. [34] demonstrated an increase in the antioxidant activities in bulbs treated with organic fertilizers. The antioxidant activities are generally related to the chemical composition of the plants in terms of the typology of antioxidant compounds. Each single compound has its own biological activity with different effects on human health [35]. Flavonoids can scavenge free radicals and can form complexes with catalytic metal ions rendering them inactive. There is also evidence of an additional mechanism by which total phenols protect against oxidative stress by producing hydrogen peroxide (H_2_O_2_), which can then help to regulate immune response actions, such as cellular growth [36].

**Figure 4:**
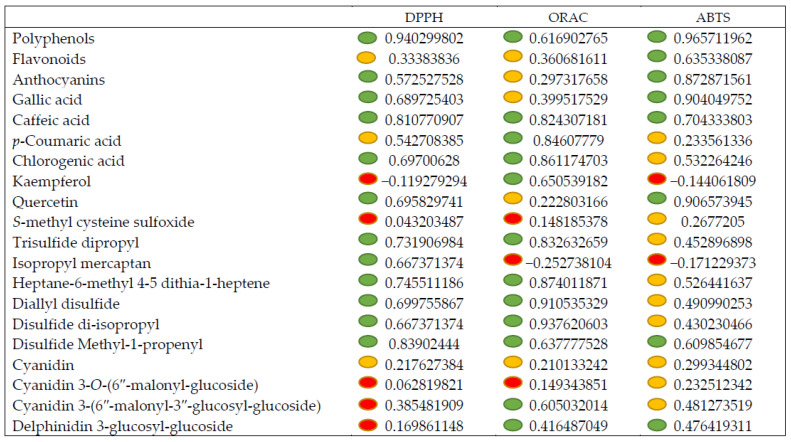
Pearson’s correlations (r) between phytochemicals and antioxidant activities. The boxed dots show the significant correlations between values; the color shows the level of correlation (yellow boxed dots *p* < 0.05 and green boxed dots *p* < 0.01). The red dots indicate a negative correlation.

Pearson’s correlation (Figure 4) evidenced that the total phenols were positively and significantly correlated with ABTS (r = 0.96), DPPH (r = 0.57), and ORAC (r = 0.62); the flavonoids correlated only with ABTS (r = 0.63), while the anthocyanins correlated with DPPH (r = 0.57) and ORAC (r = 0.87). Among the single phenolic acids, caffeic acid correlated with all the antioxidant activities, gallic acid correlated with DPPH and ABTS and chlorogenic acid with DPPH and ORAC, while *p*-coumaric acid correlated only with ORAC (r = 0.85). Among the flavonoids, the flavanol quercetin correlated with DPPH and ABTS. S-methyl cysteine sulfoxide was not involved in the antioxidative system; conversely the organic volatile compounds correlated with the antioxidant activities, mostly with DPPH and ORAC and less with ABTS.

### 2.2. Effects Red Onion Phytochemicals in Terms of Cell Proliferation or Cytotoxicity

**Figure 5:**
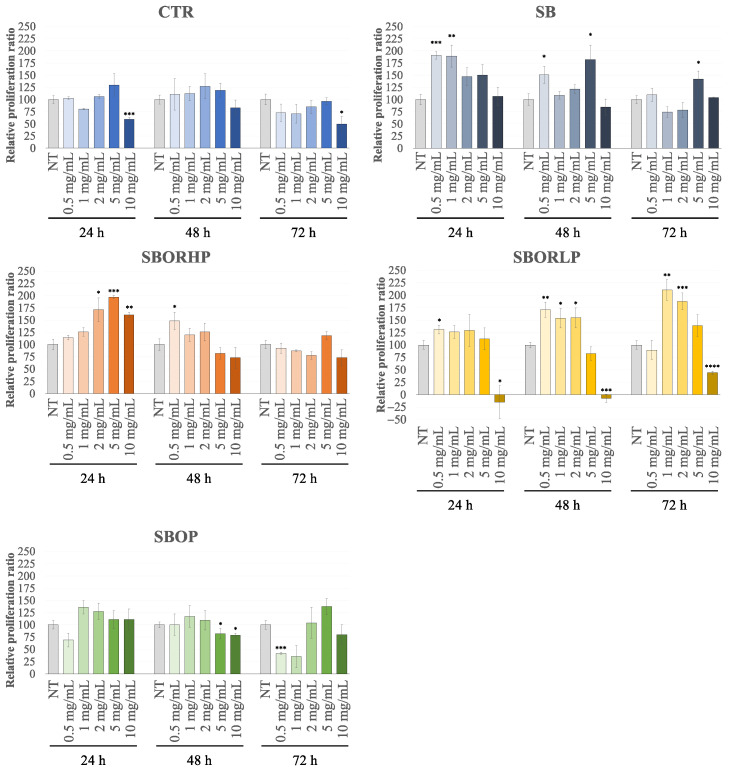
MTT assay performed on the H9c2 cell line. Cells have been treated for 24 h, 48 h and 72 h with different samples of red onions at different concentrations ranging from 0.5 mg/mL to 10 mg/mL. Data were the means ± SEM from at least 3 independent experiments under each condition and were expressed as the percentage of vehicle-treated cells. Statistical analyses were performed using Brown-Forsythe and Welch one-way analysis of variance, and mean comparisons were made using the unpaired *t*-test with Welch’s correction. * *p* < 0.05, ** *p* < 0.01, *** *p* < 0.001, and **** *p* < 0.0001.

To evaluate the possible effects of the phytochemical contents of onion samples in terms of cell proliferation or cytotoxicity, in this work, the H9c2 cells, found to be closer to normal primary cardiomyocytes for their energy metabolism features, were successfully used as an in vitro cellular model [18]. The H9c2 cells were incubated with red onion samples, fertilized, and not with the different pads in a range of concentrations between 0.5 and 10 mg/mL, and the cell viability was determined 24, 48 and 72 h after treatment following the chemical reduction of 3-(4,5-dimethylthiazol-2-yl)-2,5-diphenyltet-razolium bromide (MTT) by mitochondrial reductases in live cells [37]. As reported in Figure 5, the fertilization of red onion with recycled sulphur bentonite pads modified the red onion samples’ ability to affect the proliferation rate and/or the oxidative metabolism of H9c2 cells with respect to the ‘CTR’ one. No significant toxic effects on cell viability were detected in the different conditions for all the onion samples up to a concentration of 10 mg/mL, except for the ‘CTR’ and ‘SBORLP’ at the highest concentration. In the latter, the toxic effect was very strong, and it was already observed at 24 h of treatment. Furthermore, at 72 h of incubation time, a toxic effect was also observed with ‘SBOP’ at a low concentration.

Noteworthy is the significant increase in cell viability of H9c2 cells treated with ‘SB’, ‘SBOR LP’, and ‘SBOR HP’ samples as compared with the ‘CTR’ one, even at low concentrations and already after 24 h of treatment, which could be caused by an increase in the cells’ number and/or by an improvement in the oxidative metabolism. The effect was noticeable as early as after 24 h of treatment at very low concentrations (0.5 and 1 mg/mL) and up to 72 h for the ‘SB’ sample. ‘SBOP’ pads reduced these effects, as indicated by the overall similar results obtained with the ‘CTR’ and ‘SBOP’ treatments. Red onion samples’ capabilities to alter the proliferation rate and/or the oxidative metabolism were more evident after 24 h of treatment with ‘SBOR HP’ and after longer exposure times, 48 and 72 h, in the presence of ‘SBOR LP’. Overall, these data show a positive effect on the cell viability of H9c2, possibly related to an increase in energy metabolism, in the presence of ‘SB’ alone or with the addition of orange residue both at low and high percentages as compared to the ‘CRT’, likely due to the greatest level in bioactive compounds. To evaluate if the different red onion samples were able to protect H9c2 in oxidative stress conditions, the cells were pretreated with them for 24, 48 and 72 h before the exposure for 45 min to tert-butyl hydroperoxide (TBHP), an exogenous oxidative stress inducer. According to the treatment conditions of the previous screening, the cells were treated with two concentrations of onion samples, 0.5 and 5 mg/mL, except for ‘SBOR LP’, for which the concentrations of 1 and 5 mg/mL were used.

**Figure 6:**
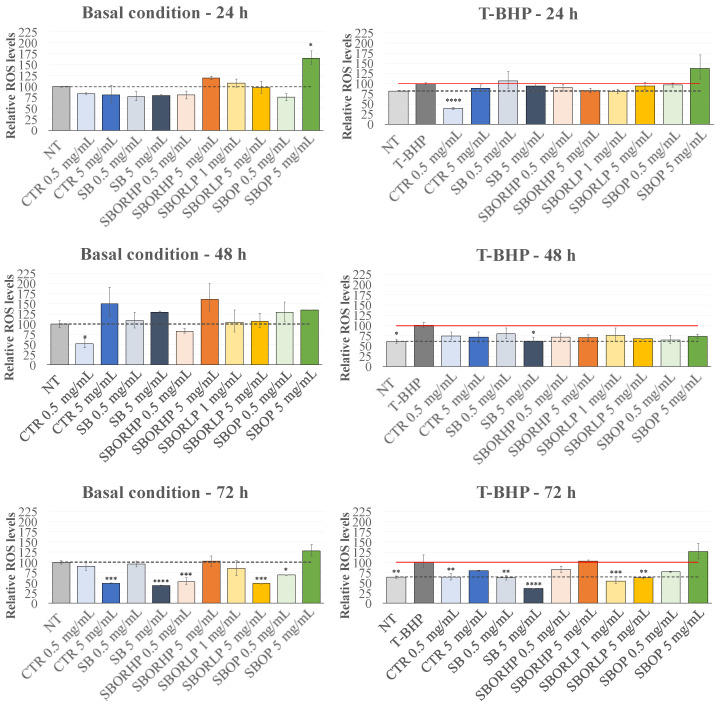
ROS detection performed on the H9c2 cell line. Cells were treated for 24 h, 48 h and 72 h with different samples of red onions at two different concentrations, and the ROS levels were measured by the DCF assay under the basal conditions and after exposure to the exogenous inducer of oxidative stress, T-BHP. Data were the, means ± SEM from at least 3 independent experiments under each condition and were expressed as the percentage of vehicle-treated cells (dotted line) or tert-Butyl hydroperoxide-treated-cells (T-BHP) (red line). Statistical analyses were performed using Brown–Forsythe and Welch one-way analysis of variance, and mean comparisons were made using the unpaired *t*-test with Welch’s correction. * *p* < 0.05, ** *p* < 0.01, *** *p* < 0.001, **** *p* < 0.0001.

Firstly, the basal ROS levels after treatment with the onion samples for 24, 48 and 72 h were measured (Figure 6). No changes were observed in the ROS levels for all onion samples, except for an increase observed in the presence of 5 mg/mL ‘SBOP’ at 24 h of incubation, which returned to the basal level already at 48 h of incubation. A significant decrease of the basal ROS levels was, however, observed at the longest incubation time, with all the onion samples, albeit at different concentrations and, in particular, in the presence of ‘SBOR HP’ and ‘SBOP’ samples, at 0.5 mg/mL. Intriguingly, these same samples showed no effects at the highest concentration. On the contrary, the ‘SB’ and ‘SBOR LP’ samples showed the same effects elicited by the ‘CTR’ red onions, inducing a decrease of the basal ROS level at a higher concentration (5 mg/mL).

Next, the effects of red onion samples were measured on the ROS levels under TBHP-induced oxidative stress conditions (Figure 6). Even in this stress condition, only the longest pretreatment with the red onion samples showed an evident effect restoring the ROS basal levels or, in the case of the highest concentration of the ‘SB’ sample, further reducing them. Notably, ‘SBORLP’ showed the same capability of reducing the ROS levels to the basal ones at both concentrations used in the pretreatment. An early significant effect, at 24 h, was observed after incubation with the lower concentration of the ‘CTR’ extract and in the presence of 5 mg/mL of ‘SB’ at 48 h. The results that the different onion samples were able to decrease TBHP-induced oxidative stress could be ascribed either to a direct scavenger activity or to an enhancement of the activity of the antioxidant defenses that neutralize the ROS levels [38].

Finally, the ‘SB’, ‘SBOR’ and ‘SBOP’ treatments influenced, even if to different extents, the H9c2 viability and oxygen radical homeostasis with respect to the ‘CTR’ sample. In particular, the ‘SBOR’ treatments showed an interesting influence on the viability of H9c2 cells, dependent on the concentration of orange residue and time of exposure, requiring longer exposure times, in some cases, when the percentage was lower. Notably, ‘SBORLP’-fertilized onions reduced the ROS levels in the basal and in oxidative stress conditions, confirming the results related to the in vitro antioxidant capacity determined by the ORAC, DPPH and ABTS assays. In particular, the results related to the oxygen radical homeostasis for the ‘SBOR’ treatments were not due only to the greatest content of the phenolic component present in these samples but also to the higher level of the organosulphides that have been shown to scavenge ROS and prevent damage caused by oxidative stress [39].

**Figure 7:**
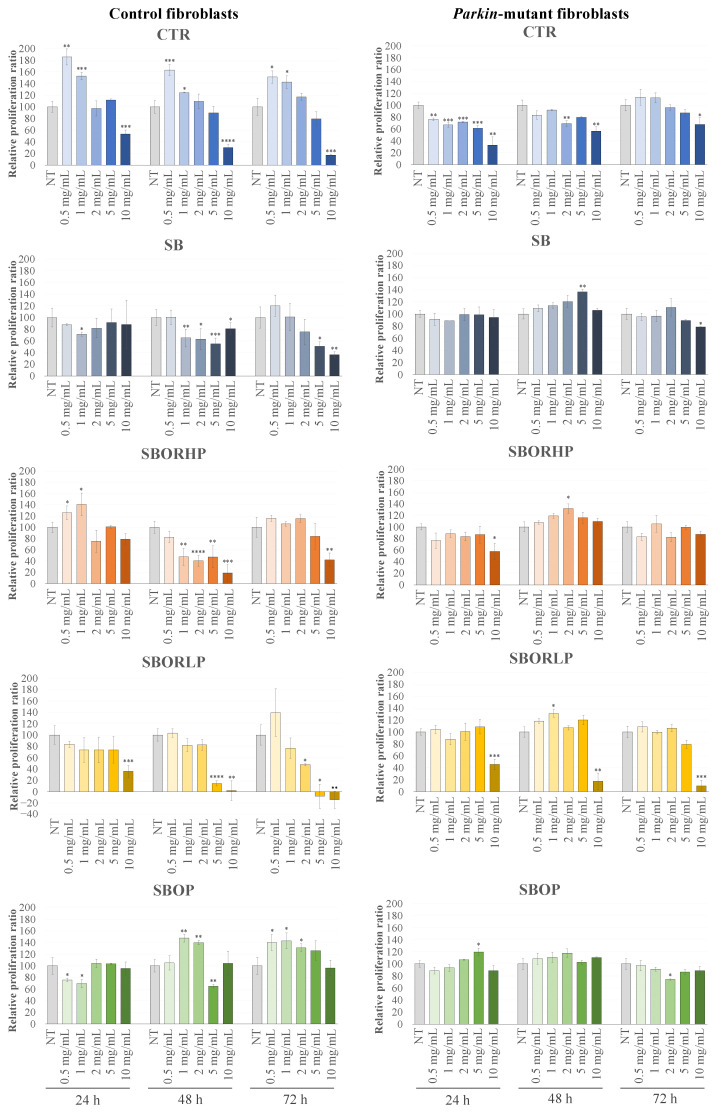
The MTT assay performed on the control and *parkin*-mutant fibroblasts. Cells were treated for 24 h, 48 h and 72 h with different samples of red onions at different concentrations ranging from 0.5 mg/mL to 10 mg/mL. Data were the means ± SEM from at least 3 independent experiments under each condition and were expressed as the percentage of vehicle-treated cells. Statistical analyses were performed using Brown-Forsythe and Welch one-way analysis of variance, and mean comparisons were made using the unpaired *t*-test with Welch’s correction. * *p* < 0.05, ** *p* < 0.01, *** *p* < 0.001, and **** *p* < 0.0001.

To assess the potential benefits of the differentially fertilized red onion samples in a pathological scenario, highly characterized primary human skin fibroblasts isolated from a healthy subject (control fibroblasts) and from a patient affected by early-onset Parkinson’s disease (*parkin*-mutant) fibroblasts [40,41,42,43,44,45,46,47,48] were treated as previously described for H9c2 treatment. Indeed, *parkin*-mutant fibroblasts are representative of oxidative stress-correlated chronic diseases, as they display mitochondrial defects associated with deregulated reactive oxygen species (ROS) production, along with impaired energy metabolism and lipid oxidation [42]. As described in Figure 7, the incubation of control fibroblasts at low concentrations of the ‘CTR’ sample (0.5 and 1 mg/mL) showed an increase in the cell viability after 24, 48 and 72 h and a gradual decrease at the highest concentrations (5 and 10 mg/mL), resulting in a significant inhibition of cell proliferation. Furthermore, an increase in cell proliferation was also observed after 24 h of treatment in the presence of low concentrations of ‘SBOR HP’ and after 48 h and 72 h in the presence of ‘SBOP’. Noteworthy, treatments with all the different onion samples at high concentrations and at long incubation times induced an inhibition of cell vitality of control fibroblasts, except for ‘SBOP’, which showed a protective action. In *parkin*-mutant fibroblasts, whereas the treatment with the ‘CTR’ sample induced a reduction in cellular viability, even at low concentrations and short incubation times, in the presence of all the other onion treatments, except for ‘SBOR LP’ at the highest concentrations, no change in the cellular vitality was observed. The lack of increase in the cellular vitality in the *parkin*-mutant fibroblasts, which was instead observed in control fibroblasts, could be due to the specific impairment of these cells. *Parkin*-mutant fibroblasts adapted to live in an environment characterized by a condition of oxidative stress showed a deficit in the mitochondrial biogenesis process, which could not lead to an increase in the cellular proliferation induced by the red onion samples. Finally, in the control fibroblasts, ‘CTR’, ‘SBOR HP’ and ‘SBOP’ onion extracts were able to increase the cellular vitality not observed in *parkin*-mutant fibroblasts. In these latter fibroblasts, the treatments with fertilized red onion samples could avoid the decrease in cellular vitality that was instead observed in the presence of the ‘CTR’ sample.

**Figure 8:**
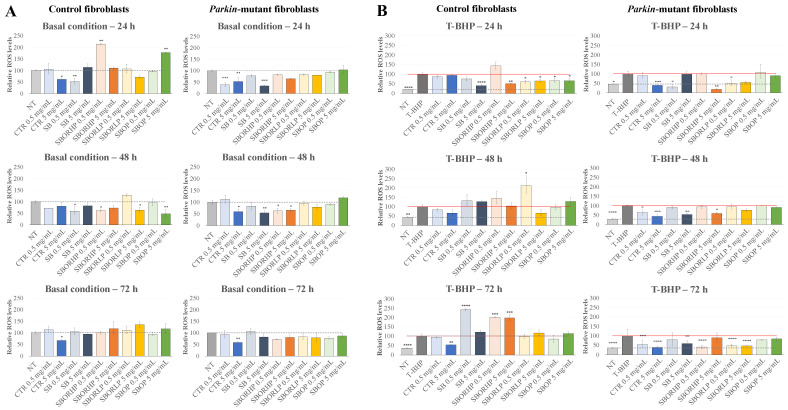
ROS detection performed on the control and *parkin*-mutant fibroblasts. Cells were treated for 24 h, 48 h, and 72 h with different samples of red onions at two different concentrations, and the ROS levels were measured by the DCF assay in the basal condition (**A**) and after exposure to the exogenous inducer of oxidative stress, T-BHP (**B**). Data were the means ± SEM from at least 3 independent experiments under each condition and were expressed as a percentage of vehicle-treated cells (dotted line) (**A**) or tert-Butyl hydroperoxide-treated cells (T-BHP) (red line) (**B**). Statistical analyses were performed using one-way analysis of variance, and mean comparisons were made using Fisher’s LSD test. * *p* < 0.05, ** *p* < 0.01, *** *p* < 0.001, and **** *p* < 0.0001.

As described previously for the H9c2 cell line, it was evaluated if the different red onion samples were able to protect human fibroblasts in TBHP-induced oxidative stress conditions. According to the findings of the viability screening, cells were treated with 0.5 and 5 mg/mL for 24, 48 and 72 h (Figure 8A). In control fibroblasts, a decrease in the basal ROS levels was observed already at 24 h of incubation in the presence of high concentrations of ‘CTR’ but low concentrations of ‘SB’ samples. Furthermore, a decrease in the ROS basal levels was induced by ‘SB’ at 48 h of incubation, as well as by ‘SBOR HP’, ‘SBOR LP’, and at a higher extent, by ‘SBOP’. It is possible to assume that the increase in the ROS basal level observed at 24 h of incubation in the presence of 0.5 mg/mL and 5 mg/mL of ‘SBOR HP’ and ‘SBOP’, respectively, might have induced an antioxidant enzymatic response, which, in turn, resulted in a ROS scavenger effect at 48 h of incubation. In *parkin*-mutant fibroblasts at 24 h of incubation, the ‘CTR’ induced a decrease at the basal ROS levels at low and high concentrations, and this effect persisted also at the highest concentrations and longest incubation times, similar to what was observed in the control cells. In addition, the decrease in the basal ROS levels was observed at 24 and 48 h of incubation in the presence of 5 mg/mL of ‘SB’ and at low and high concentrations of ‘SBORHP’ after 48 h of treatment. The scavenger effect of the ‘CTR’ sample, observed in *parkin*-mutant fibroblasts, especially in oxidative stress conditions, demonstrated the effectiveness of red onion already rich in bioactive compounds.

Next, the effects of red onion samples on the ROS levels under TBHP-induced oxidative stress conditions were assessed (Figure 8B). In this condition, the behavior observed in the control and *parkin*-mutant fibroblasts was substantially different, mainly the long incubation time. A significant decrease was observed in the control cells at 24 h of incubation with all the treated onion samples, albeit at different concentrations, as compared with the ‘CTR’. In *parkin*-mutant fibroblasts in the same conditions, a reduction of TBHP-induced oxidative stress was observed at higher concentrations of the ‘CTR’ and in the presence of ‘SB’, ‘SBOR HP’ and ‘SBOR LP’ samples. At 48 and 72 h of incubation, no effect of red onion treatment on TBHP-induced oxidative stress was observed in the control cells, except for the reduction of the ROS level in the presence of a high concentration of ‘CTR’ over long time of incubation, whereas a reduction at 48 h of incubation was observed in *parkin*-mutant fibroblasts in the presence of a different concentration of ‘CTR’, ‘SB’ and ‘SBOR HP’ samples, as well as at 72 h with low and high concentrations of ‘SBOR LP’. Furthermore, in the control cells, an increase in the ROS level with respect to TBHP-induced oxidative stress was observed with 0.5 mg/mL of ‘SB’ and with low and high concentrations of ‘SBOR HP’, highlighting a possible toxic effect, as already shown by the decrease in cellular viability in this condition. The scavenger effect observed in *parkin*-mutant fibroblasts and in basal and TBHP-induced oxidative stress conditions pointed out an effective protective role of red onion samples fertilized with sulphur bentonite containing orange, particularly at low concentrations with respect to the ‘CTR’ sample.

## 3. Materials and Methods

### 3.1. Chemicals

Metaphosphoric acid, 2,2-diphenyl-1-picrylhydrazyl (DPPH),NaOH, nitroblue tetrazolium, dichlorophenol-indophenol (DCPID),2,2′-azino-bis (3-ethylbenzothiazoline-6-sulfonic acid) di-ammoniumsalt (ABTS•+), 6-hydroxy-2,5,7,8-tetramethylchromane-2-carboxyl acid(Trolox), phenazine methosulphate, ethanol, gallic acid, ethylenediaminetetraaceticacid (EDTA), ferrozine, 2,4,6-tris(2-piridil)-s-triazina(TPTZ) and iron sulphate heptahydrate were purchased from Sigma Chemical Co. (St. Louis, MO, USA). Acetonitrile and acetic acid were HPLC-grade and were purchased from Merck (Darmstadt, Germany). All the phenolic standards were obtained from Extra Syntheses (Genay, France). Solvents and reagents for carotenoid detection were purchasedfrom Panreac (Barcelona, Spain). Other chemicals were of analyticalgrade purchased from Carlo Erba Reagents s.r.l. (Cornaredo, MI, Italy).

### 3.2. Red Onion Experimental Conditions

The experiment was conducted in triplicate for 3 months in the field (until the bulb is fully ripe) in alkaline sandy-loam soil with a pH of 8.5. The soils contained 3.09% organic matter, 0.17% nitrogen, 110 g∙kg^−1^ CaCO_3_ and 0.334 g∙kg^−1^ SO_4_. In each parcel, 30 uniform seedlings of red onion/m squared were transplanted. The soil was divided into parcels of 10 m squared. In each parcel, 30 uniform seedlings of red onion/m^2^ were transplanted. Pads of sulphur bentonite (SB, 90%/10%) or SB with two percentages of orange residue (SBOR) conventionally called high (with a greater amount of orange residue, SBORHP) and low (with a lower amount of orange residue, SBORLP) or SBOP with olive pomace (OP) were used at a concentration of 16 gm^−2^ (corresponding to 476 kg S ha^−1^), the dose generally used to lower the pH and to replenish the S. Sulphur was the major component of the fertilizers. The industrial process and the formulation of the pads are covered by an industrial secret drawn up in the agreement signed with Steel Belt System in 2015. Nonamended soils were used as the control CTR (Table 3). OP was used only at a low concentration, because previous experiments (data not shown) demonstrated a toxicity of OP on crops increasing its concentration (preliminary experiments carried out in a greenhouse, data not shown). SB and SB with OP or OR produced onions with greater bulb sizes with respect to the control (data not shown).

During the experiment, all onion plants were regularly irrigated to maintain 70% of their field capacity. At harvest time (3 months), onions were collected and stored at −20 °C for chemical and biological determinations.

### 3.3. Extraction and Determination of Total Anthocyanins

The assessment of the total anthocyanin content was carried out by the pH differential method according to the Official Methods of Analysis of AOAC International, as previously described by Muscolo et al. [17]. Absorbance was measured using a 1800 UV-Vis Spectrophotometer (Shimadzu, Kyoto, Japan) at 510 and 700 nm in buffers at pH 1.0 and 4.5. Values were expressed as mg cyanidin-3-glucoside equivalent g^−1^ dry weight (DW) using 26,900 as the molar extinction coefficient.

### 3.4. Ethanolic Extracts

Five hundred milligrams of frozen onion samples were weighed and extracted at room temperature under continuous stirring for 1.5 h with ethanol (15 mL), as described in Muscolo et al. [17]. The samples were centrifuged at 2365× *g* for 15 min, and the supernatants were filtered dried and resuspended in 3 mL of ethanol.

### 3.5. Determination of Total Phenolic Compounds and Total Flavonoids

The Folin–Ciocalteu assay was used for evaluating the total phenol content as reported in Muscolo et al. [17]. The absorbance of the samples was recorded at 760 nm. A calibration curve was constructed with gallic acid, and the results were expressed as the gallic acid equivalent (GAE) in mg∙g^−1^ DW.

The total flavonoid was detected according to the spectrophotometric method, as reported in Muscolo et al. [17]. One milliliter of extract was mixed with 1 mL of 20 g L^−1^ AlCl_3_ methanolic solution. After incubation at room temperature for 15 min, the absorbance was measured at 430 nm. The flavonoid content was calculated from a calibration curve of rutin and expressed as mg rutin g^−1^ DW.

### 3.6. Determination of Antioxidant Activities

The antioxidant activity against the DPPH (2,2-diphenyl-1-picryl-hydrazyl-hydrate) radical was determined according to [49]. The DPPH concentration in the cuvette was chosen to give absorbance values of ≈1.0. The reaction mixtures were composed of 10 μL of each extract, 700 μL of DPPH, and ethanol up to a final volume of 1 mL. A blank without ethanol extract was prepared for each sample. The change in absorbance of the violet solution was recorded at 517 nm after 30 min of incubation at 37 °C. The inhibition I (%) of radical scavenging activity was calculated as I (%) = [(A0 − AS)/A0] × 100, where A0 is the absorbance of the control, and AS is the absorbance of the sample after 30 min of incubation. The results were expressed as Trolox equivalents (TE).

The ABTS (2,2′-Azino-bis(3-ethylbenzothiazoline-6-sulfonic acid) assay was performed according to [50]. Solutions of 7 mmol L^−1^ ABTS^+^ (final concentration) and 2.45 mmol L^−1^ ammonium persulfate (final concentration) in phosphate-buffered saline (PBS) were mixed and kept in the dark at room temperature for 12–16 h. Before use, the absorbance of the ABTS^+^ solution was fixed at 0.70 ± 0.02 at 734 nm. Aliquots of ethanol extract (25, 50 and 100 μL) were added to 0.5 mL of ABTS^+^ solution and brought to a final volume of 600 μL with PBS. After 6 min of incubation in the dark at room temperature, the absorbance of the samples was recorded at 734 nm using a UV-Visible spectrophotometer. The inhibition I (%) of radical scavenging activity was calculated as I (%) = [(A0 − AS)/A0] × 100, where A0 is the absorbance of the control, and AS is the absorbance of the sample after 4 min of incubation. The results were expressed as μmol∙L^−1^ TE using a Trolox (1–50 μmol∙L^−1^) calibration curve.

The oxygen radical absorbance capacity (ORAC) assay was performed according to [50]. A 20-μL aliquot of extract was added to 120 μL of fresh fluorescein solution (117 nmol L^−1^). After a preincubation time of 15 min at 37 °C, 60 μL of freshly prepared AAPH solution (40 mmol L^−1^) was added. Fluorescence was recorded every 30 s for 90 min (𝜆ex 485 nm, 𝜆em 520 nm). A blank using 20 μL of methanol instead of the sample was also analyzed. ORAC values were expressed as μmol TE mg^−1^ FW using a Trolox (10–100 μmol L^−1^) calibration curve.

### 3.7. HPLC and Gas Chromatography/Mass Spectrometry (GC/MS) Analysis of Volatile Organic Compounds

Frozen onion samples (1 g) were incubated overnight in absolute methanol at 4 °C. Then, the methanol was separated from the pieces of onion and collected in a balloon. The onion pieces were homogenized with absolute methanol (10 mL) in a mortar and stirred 30 min at room temperature (25 °C). Samples were then centrifuged, and each supernatant was mixed with the other methanol. The precipitates were resuspended in methanol (10 mL), and the above operations were repeated twice. The methanolic phases were combined, reduced to a volume of 10 mL in a rotary evaporator, and stored at −18 °C until use. Methanolic extracts (1 mL) were diluted with dimethylformamide (1 mL) and filtered through an Iso-Disk P-34, 3 mm in diameter poly(tetrafluoroethylene) (PTFE) membrane, and 0.45 μm pore size supplied by Supelco. Diode array detection (DAD)-HPLC (Shimadzu, Kyoto, Japan) separation of onion flavonoids was performed according to the method described by [49].

Reverse phase-diode array detector-high-performance liquid chromatography (RP-DAD-HPLC) analyses of the samples were carried out with a Shimadzu system (Kyoto, Japan) consisting of a LC-10AD pump system, a vacuum degasser, a quaternary solvent mixing, a SPD-M10 diode array detector, and a Rheodyne 7725i injector (Merck KGaA, Darmstadt, Germany). Separation of each compound was done on a 250 × 4.6 mm i.d., 5-μm Discovery C18 column, supplied by Supelco Park (Bellefonte, PA, USA) equipped with a 4.0 × 20-mm guard column. The column was placed in a column oven set at 25 °C. The injection loop was 20 μL, and the flow rate was 1.0 mL/min. The mobile phase consisted of a linear gradient of solvent A (acetonitrile) in 2% acidified water (acetic acid:H_2_O, 2:98) as follows: 0–80% (0–55 min), 90% (55–70 min), 95% (70–80 min), 100% (80–90 min), and 0% (90–110 min). UV-Vis spectra were measured between 200 and 600 nm and simultaneous detection using a diode array at 278 and 325 nm. Compounds were identified using their retention time and UV spectra through comparisons with purified standards (Sigma Chemical Co., St. Louis, MO, USA). Anthocyanins were extracted from frozen onion tissues (0.5 g) homogenized in a mortar with 10 mL of methanol containing1 mL L^−1^ HCl at room temperature for 2 h. The extracts were filtered through an Iso-Disk P-34, 3 mm in diameter PTFE membrane of 0.45-μm pore size (Supelco), and utilized for HPLC analysis. HPLC separation was carried out using a Spherisorb S5 ODS2, Merck KGaA, Darmstadt, Germany (250 mm × 4.6 mm i.d., 5 μm), as described by [51]. To detect S-methyl-l-cysteine sulfoxide (SMCSO), small pieces of frozen red onion (250 mg) were homogenized with 5 mL of distilled water and filtered through filter paper. SMCSO was quantified by HPLC after derivatization with o-phthalaldehyde, as reported in [52].

A red onion volatile organic compound (VOC) analysis was performed using a Thermo Fisher gas chromatograph (TRACE 1310, Thermo Fisher Scientific, Waltham, MA, USA) equipped with a single-quadrupole mass spectrometer (ISQ LT, Thermo Fisher Scientific, Waltham, MA, USA), as reported in [17].

### 3.8. Red Onion Sample Preparation for Cell Culture Treatments

The lyophilized onion samples were dissolved in high-glucose Dulbecco’s modified Eagle’s medium (DMEM) supplemented with 10% (*v*/*v*) fetal bovine serum (FBS), 1% (*v*/*v*) L-glutamine, and 1% (*v*/*v*) penicillin/streptomycin and incubated for 1 h at 37 °C. After this time, samples were centrifuged at 2600× *g* for 15 min, and the supernatant was filtered and sterilized through a 0.22-µm membrane filter. The final concentration of the stock solution was 10 mg/mL in DMEM. The onion extract (OE) concentrations tested for the cell culture treatments were: 0.5, 1, 2, 5 and 10 mg/mL.

### 3.9. Cells and Culture Conditions

The H9c2 cell line, derived from embryonic rat hearts (ATCC; Manassas, VA, USA), and primary fibroblasts from a patient affected by early-onset Parkinson’s disease (PD) and from one healthy subject, obtained by explants from a skin punch biopsy after informed consent [40,41], were grown in high-glucose Dulbecco’s modified Eagle’s medium (DMEM) supplemented with 10% (*v*/*v*) fetal bovine serum (FBS), 1% (*v*/*v*) l-glutamine, and 1% (*v*/*v*) penicillin/streptomycin at 37 °C in a humidified atmosphere of 5% CO_2_. For the treatment conditions, cells were seeded in 96-well plates and grown for 24 h. After that, the media was removed, and the cells were cultured at 37 °C in culture plates for 24, 48, and 72 h in fresh media containing different concentrations of red onion samples.

### 3.10. Cell Viability

Cell viability was assessed by the 3-(4,5-dimethylthiazol-2-yl)2,5-diphenyltetrazolium (MTT) assay after 24, 48 and 72 h of exposure of the cells and seeded in 96-well plates to the different concentrations of red onion samples. After the incubation, 150 µL DMEM and 15 µL of MTT (5 mg/mL) were added to each well. The plates were incubated for 3 h at 37 °C. The media was removed, and formazan crystals were dissolved in 150 µL of isopropanol with gentle shaking. The absorbance was measured at 570 nm by the Victor 2030 multilabel reader (PerkinElmer, Waltham, MA, USA).

### 3.11. Determination of Reactive Oxygen Species (ROS)

The H_2_O_2_ levels were determined by the cell permeant probe 2′-7′-dichlorodihydrofluorescin diacetate (H_2_DCFDA). Briefly, after 24, 48 and 72 h of exposure to specific red onion sample concentrations (0, 0.5, 1 and 5 mg/mL), based on the results of the cell viability assay, the media were changed and oxidative stress was induced by 50 µM tert-butyl hydroperoxide (T-BHP) treatment (Sigma-Aldrich, B2633, St. Louis, MO, USA), as described in [53,54]. After 45 min of treatment, the cells were incubated in the dark, at 37 °C for 20 min with 10 µM H_2_DCFDA. After that, the cells were washed and resuspended in 150 µL of PBS, and the H_2_O_2_-dependent oxidation of the fluorescent probe was measured by the Victor 2030 multilabel reader (PerkinElmer, Waltham, MA, USA) (at 507-nm excitation and 530-nm emission wavelength).

### 3.12. Statistical Analysis

Analysis of variance was carried out for all the data sets. One-way ANOVA with Tukey’s honestly significant difference test was carried out to analyze the effects of fertilizers on each of the various parameters measured. The ANOVA and *t*-test were carried out using SPSS software (IBM Corp. 2012). The effects were significant at *p* ≤ 0.05.

Raw data from MTT assays and ROS determinations were first analyzed in Microsoft Excel Spreadsheet software to calculate the relative proliferation and ROS levels ratios, respectively. Then, log-transformed data were imported to GraphPad Prism to apply statistical tests. The Welch and Brown–Forsythe versions of one-way ANOVA were used to compare samples treated with different red onions at different concentrations, while each comparison was evaluated by applying an unpaired *t*-test with Welch’s correction. Statistical significance was set at *p* < 0.05.

## 4. Conclusions

These intriguing results may be explained by the different bioactive compounds identified in each fertilized red onion. SBOR onion, which showed the best positive effects on the cell culture treatment, contained the highest amount of total phenols, single phenolic acids, kaempferol, anthocyanidins, S-methyl cysteine sulfoxide, and volatile compounds correlated to a better in vitro antioxidant capacity as determined by the DPPH, ABTS and ORAC assays. The correlation data evidenced a diversity of action, at the metabolic level, of the different classes of secondary metabolites and mostly of the single compounds belonging to the different classes, evidencing that the chemical structure of a biocompound can determines its reactivity versus free radicals and other ROS, influencing the antioxidant activity.

Considering the data of the relative cellular vitality, the positive effects could be due to the total phenols and, in particular, to the great presence of specific phenolic acids such as chlorogenic and *p*-coumaric and, also, to the flavanol kaempferol, which were more correlated with ORAC and more present in red onions treated with SBOR(LP) than in the other fertilized red onions. In addition to their great ability as scavengers, free phenolic acids, unlike flavonoids, have a high bioavailability and good water solubility [55] and can be absorbed in the stomach, contrary to flavonoids that cannot be absorbed, and only their small quantity can be transported passively through the intestinal wall into the blood [56].

Our study describes, for the first time, the antioxidant effect of the bioactive phenolic fraction from red onion bulbs fertilized with sulphur-bentonite enriched with orange residue or olive pomace on rat cardiomyocytes and primary human fibroblasts. This work is a pilot study that highlighted significant and useful data of how sustainable fertilization can lead to the improvement of the quality of red onions that can be used to develop functional foods or nutraceuticals for the prevention and management of numerous diseases.

From this manuscript emerges how the use of sulphur-bentonite-based fertilizers can represent a tool to increase, also in other species, phytochemicals with beneficial effects on human health and how the antioxidant activity/capacity of functional foods is important in preventing and treating numerous diseases. The phytochemicals enhance the medical and economic values of crops with important consequences on the bio and green economy, creating new opportunities for business.

Further investigations are in progress to test the effects on the H9c2 rat cardiomyoblast cell line and primary human dermal fibroblasts, in terms of viability and oxygen radical homeostasis mammalian cells, of each single compound identified in the extracts, with the aim to verify if the positive effects are due to a single specific compound or to a synergic or additive effect of more compounds.

## Figures and Tables

**Table 1:** Total phenols (mg∙GAE∙g^−1^ DW), flavonoids (mg∙rutin∙g^−1^ DW), and anthocyanins (mg∙cyanidin-3-glucosideg^−1^ DW) found in red onion bulbs differently fertilized: control (CTR), sulphur bentonite (SB), sulphur bentonite-low percentage orange residue (SBOR LP), sulphur bentonite-high percentage orange residue (SBOR HP), and sulphur bentonite-olive pomace (SBOP). Data are the mean of three replicates ± the standard error.

	Total Phenols	Flavonoids	Anthocyanins
**CTR**	28 ± 1 ^c^	2.1 ± 0.1 ^c^	23 ± 1 ^b^
**SB**	37 ± 2 ^b^	3.7 ± 0.2 ^b^	37 ± 2 ^a^
**SBORLP**	48 ± 2 ^a^	3.9 ± 0.3 ^b^	37 ± 2 ^a^
**SBORHP**	43 ± 3 ^a^	3.8 ± 0.2 ^b^	37 ± 2 ^a^
**SBOP**	37 ± 2 ^b^	5.0 ± 0.5 ^a^	37 ± 2 ^a^

Means followed by different letters in the same column are significantly different (Tukey’s test at *p* < 0.05).

**Table 2:** S-Methyl-l-cysteine sulfoxide (μg∙g^−1^ FW) and the relative concentration μg∙g^−1^ FW of volatile organic compounds in the onion bulbs differently fertilized: control (CTR), sulphur bentonite (SB), sulphur bentonite-low percentage orange residue (SBOR LP), sulphur bentonite-high percentage orange residue (SBOR HP), and sulphur bentonite-olive pomace (SBOP). Data are the mean of three replicates ± the standard error.

ID	S-methyl Cysteine Sulfoxide	Trisulfide Dipropyl	Diallyl Disulfide	Disulfide Di-Isopropyl	Disulfide Methyl-l-Propenyl	Isopropyl Mercaptan	Heptane-6-Methyl 4-5 Dithia-1-Heptene
CTR	110 ± 9 ^e^	3.32 ± 0.5 ^b^	nd	36.7 ± 2 ^b^	0.38 ± 0.04 ^c^	53 ± 3 ^b^	1.0 ± 0.2 ^c^
SB	590 ± 12 ^a^	1.95 ± 0.2 ^c^	nd	34.3 ± 3 ^b^	nd	61 ± 4 ^a^	1.0 ± 0.1 ^c^
SBORLP	390 ±13 ^b^	10.3 ± 1 ^a^	0.32 ± 9 ^a^	53.6 ± 2 ^a^	1.44 ± 0.05 ^a^	53 ± 2 ^b^	8.4 ± 1 ^a^
SBORHP	140 ± 12 ^c^	3.32 ± 0.6 ^b^	nd	35.3 ± 1 ^b^	0.73 ± 0.02 ^b^	51 ± 3 ^b^	1.5 ± 0.2 ^b^
SBOP	180 ± 12 ^d^	1.63 ± 0.5 ^e^	nd	36.7 ± 3 ^b^	0.07 ± 0.001 ^d^	54 ± 2 ^b^	0.6 ± 0.02 ^d^

Means followed by different letters in the same column are significantly different (Tukey’s test at *p* < 0.05).

**Table 3:** Experimental design.

ID Sample	Description
CTR	Control unfertilized soil
SB	Onion grown with sulphur bentonite
SBOR HP	Onion grown with sulphur bentonite-high percentage of orange residue
SBOR LP	Onion grown with sulphur bentonite-low percentage of orange residue
SBOP	Onion grown with sulphur bentonite and olive pomace

## Data Availability

Not applicable.

## Sample Availability

Samples of the compounds are available from all the authors.

## References

[1] Finkel T., Holbrook N.J. (2000). Oxidants, Oxidative Stress and the Biology of Ageing. Nature.

[2] Hajam Y.A., Rani R., Ganie S.Y., Sheikh T.A., Javaid D., Qadri S.S., Pramodh S., Alsulimani A., Alkhanani M.F., Harakeh S. (2022). Oxidative Stress in Human Pathology and Aging: Molecular Mechanisms and Perspectives. Cells.

[3] Borek C. (2001). Antioxidant Health Effects of Aged Garlic Extract. J. Nutr..

[4] Colina-Coca C., González-Peña D., De Ancos B., Sánchez-Moreno C. (2017). Dietary Onion Ameliorates Antioxidant Defence, Inflammatory Response, and Cardiovascular Risk Biomarkers in Hypercholesterolemic Wistar Rats. J. Funct. Foods.

[5] Jini D., Sharmila S. (2020). Green Synthesis of Silver Nanoparticles from *Allium cepa* and Its in Vitro Antidiabetic Activity. Mater. Today Proc..

[6] Tsuboki J., Fujiwara Y., Horlad H., Shiraishi D., Nohara T., Tayama S., Motohara T., Saito Y., Ikeda T., Takaishi K. (2016). Onionin A Inhibits Ovarian Cancer Progression by Suppressing Cancer Cell Proliferation and the Protumour Function of Macrophages. Sci. Rep..

[7] Yang E.-J., Kim G.-S., Kim J.A., Song K.-S. (2013). Protective Effects of Onion-Derived Quercetin on Glutamate-Mediated Hippocampal Neuronal Cell Death. Pharmacogn. Mag..

[8] Zhou Y., Zhuang W., Hu W., Liu G.-J., Wu T.-X., Wu X.-T. (2011). Consumption of Large Amounts of Allium Vegetables Reduces Risk for Gastric Cancer in a Meta-Analysis. Gastroenterology.

[9] Nicastro H.L., Ross S.A., Milner J.A. (2015). Garlic and Onions: Their Cancer Prevention Properties. Cancer Prev. Res. Phila. Pa.

[10] Hertog M., Katan M. (1998). Quercetin in Foods, Cardiovascular Disease, and Cancer. Flavonoids Health Dis..

[11] Hatono S., Jimenez A., Wargovich M.J. (1996). Chemopreventive Effect of S-Allylcysteine and Its Relationship to the Detoxification Enzyme Glutathione S-Transferase. Carcinogenesis.

[12] Fukushima S., Takada N., Wanibuchi H., Hori T., Min W., Ogawa M. (2001). Suppression of Chemical Carcinogenesis by Water-Soluble Organosulphur Compounds. J. Nutr..

[13] Richter M., Ebermann R., Marian B. (1999). Quercetin-Induced Apoptosis in Colorectal Tumor Cells: Possible Role of EGF Receptor Signaling. Nutr. Cancer.

[14] Miodini P., Fioravanti L., Fronzo G.D., Cappelletti V. (1999). The Two Phyto-Oestrogens Genistein and Quercetin Exert Different Effects on Oestrogen Receptor Function. Br. J. Cancer.

[15] Marefati N., Ghorani V., Shakeri F., Boskabady M., Kianian F., Rezaee R., Boskabady M.H. (2021). A Review of Anti-Inflammatory, Antioxidant, and Immunomodulatory Effects of *Allium cepa* and Its Main Constituents. Pharm. Biol..

[16] Kazimierczak R., Średnicka-Tober D., Barański M., Hallmann E., Góralska-Walczak R., Kopczyńska K., Rembiałkowska E., Górski J., Leifert C., Rempelos L. (2021). The Effect of Different Fertilization Regimes on Yield, Selected Nutrients, and Bioactive Compounds Profiles of Onion. Agronomy.

[17] Muscolo A., Papalia T., Settineri G., Mallamaci C., Panuccio M. (2019). Sulphur Bentonite-organic-based Fertilizers as Tool for Improving Bio-compounds with Antioxidant Activities in Red Onion. J. Sci. Food Agric..

[18] Kuznetsov A.V., Javadov S., Sickinger S., Frotschnig S., Grimm M. (2015). H9c2 and HL-1 Cells Demonstrate Distinct Features of Energy Metabolism, Mitochondrial Function and Sensitivity to Hypoxia-Reoxygenation. Biochim. Biophys. Acta.

[19] Rababa’h A.M., Guillory A.N., Mustafa R., Hijjawi T. (2018). Oxidative Stress and Cardiac Remodeling: An Updated Edge. Curr. Cardiol. Rev..

[20] Wei Z., Li X., Li X., Liu Q., Cheng Y. (2018). Oxidative Stress in Parkinson’s Disease: A Systematic Review and Meta-Analysis. Front. Mol. Neurosci..

[21] Ren F., Reilly K., Kerry J.P., Gaffney M., Hossain M., Rai D.K. (2017). Higher Antioxidant Activity, Total Flavonols, and Specific Quercetin Glucosides in Two Different Onion (*Allium cepa* L.) Varieties Grown under Organic Production: Results from a 6-Year Field Study. J. Agric. Food Chem..

[22] Hallmann E., Rembialkowska E. (2006). Antioxidant compounds content in selected onion bulbs from organic and conventional cultivation. J. Res. Appl. Agric. Eng. Pol..

[23] Fredotović Ž., Šprung M., Soldo B., Ljubenkov I., Budić-Leto I., Bilušić T., Čikeš-Čulić V., Puizina J. (2017). Chemical Composition and Biological Activity of *Allium Cepa* L. and *Allium* × *Cornutum* (Clementi Ex Visiani 1842) Methanolic Extracts. Molecules.

[24] Aguiar J., Gonçalves J.L., Alves V.L., Câmara J.S. (2020). Chemical Fingerprint of Free Polyphenols and Antioxidant Activity in Dietary Fruits and Vegetables Using a Non-Targeted Approach Based on QuEChERS Ultrasound-Assisted Extraction Combined with UHPLC-PDA. Antioxidants.

[25] Žilić S., Šukalović V.H.-T., Dodig D., Maksimović V., Maksimović M., Basić Z. (2011). Antioxidant Activity of Small Grain Cereals Caused by Phenolics and Lipid Soluble Antioxidants. J. Cereal Sci..

[26] Kiokias S., Proestos C., Oreopoulou V. (2020). Phenolic Acids of Plant Origin—A Review on Their Antioxidant Activity In Vitro (O/W Emulsion Systems) Along with Their in Vivo Health Biochemical Properties. Foods.

[27] Roychoudhury S., Sinha B., Choudhury B.P., Jha N.K., Palit P., Kundu S., Mandal S.C., Kolesarova A., Yousef M.I., Ruokolainen J. (2021). Scavenging Properties of Plant-Derived Natural Biomolecule Para-Coumaric Acid in the Prevention of Oxidative Stress-Induced Diseases. Antioxidants.

[28] Zhu H., Liang Q.H., Xiong X.G., Wang Y., Zhang Z.H., Sun M.J., Lu X., Wu D. (2018). Anti-Inflammatory Effects of p-Coumaric Acid, a Natural Compound of *Oldenlandia diffusa*, on Arthritis Model Rats. Evid. Based Complement. Altern. Med..

[29] Nasr Bouzaiene N., Kilani Jaziri S., Kovacic H., Chekir-Ghedira L., Ghedira K., Luis J. (2015). The effects of caffeic, coumaric and ferulic acids on proliferation, superoxide production, adhesion and migration of human tumor cells in vitro. Eur. J. Pharmacol..

[30] Alam M., Ahmed S., Elasbali A.M., Adnan M., Alam S., Hassan M.I., Pasupuleti V.R. (2022). Therapeutic Implications of Caffeic Acid in Cancer and Neurological Diseases. Front. Oncol..

[31] Silva dos Santos J., Gonçalves Cirino J.P., de Oliveira Carvalho P., Ortega M.M. (2021). The Pharmacological Action of Kaempferol in Central Nervous System Diseases: A Review. Front. Pharmacol..

[32] Omar S.H., Al-Wabel N.A. (2010). Organosulfur compounds and possible mechanism of garlic in cancer. Saudi Pharm. J..

[33] Yang L., Wen K.-S., Ruan X., Zhao Y.-X., Wei F., Wang Q. (2018). Response of Plant Secondary Metabolites to Environmental Factors. Molecules.

[34] Cavalheiro T.R.T., de Alcoforado R.O., de Silva V.S.A., Coimbra P.P.S., de Mendes N.S., Cavalcanti E.D.C., de Jurelevicius D.A., de Gonçalves É.C.B.A. (2021). The Impact of Organic Fertilizer Produced with Vegetable Residues in Lettuce (*Lactuca sativa* L.) Cultivation and Antioxidant Activity. Sustainability.

[35] Yashin A., Yashin Y., Xia X., Nemzer B. (2017). Antioxidant Activity of Spices and Their Impact on Human Health: A Review. Antioxidants.

[36] Li Q., Qiu Z., Wang Y., Guo C., Cai X., Zhang Y., Liu L., Xue H., Tang J. (2021). Tea Polyphenols Alleviate Hydrogen Peroxide-Induced Oxidative Stress Damage through the Mst/Nrf2 Axis and the Keap1/Nrf2/HO-1 Pathway in Murine RAW264.7. Cells. Exp. Ther. Med..

[37] van de Loosdrecht A.A., Beelen R.H., Ossenkoppele G.J., Broekhoven M.G., Langenhuijsen M.M. (1994). A Tetrazolium-Based Colorimetric MTT Assay to Quantitate Human Monocyte Mediated Cytotoxicity against Leukemic Cells from Cell Lines and Patients with Acute Myeloid Leukemia. J. Immunol. Methods.

[38] Mete R., Oran M., Topcu B., Oznur M., Seber E.S., Gedikbasi A., Yetisyigit T. (2016). Protective Effects of Onion (*Allium cepa*) Extract against Doxorubicin-Induced Hepatotoxicity in Rats. Toxicol. Ind. Health.

[39] Imai J., Ide N., Nagae S., Moriguchi T., Matsuura H., Itakura Y. (1994). Antioxidant and Radical Scavenging Effects of Aged Garlic Extract and Its Constituents. Planta Med..

[40] Ferretta A., Gaballo A., Tanzarella P., Piccoli C., Capitanio N., Nico B., Annese T., Di Paola M., Dell’Aquila C., De Mari M. (2014). Effect of Resveratrol on Mitochondrial Function: Implications in Parkin-Associated Familiar Parkinson’s Disease. Biochim. Biophys. Acta BBA Mol. Basis Dis..

[41] Tanzarella P., Ferretta A., Barile S.N., Ancona M., De Rasmo D., Signorile A., Papa S., Capitanio N., Pacelli C., Cocco T. (2019). Increased Levels of CAMP by the Calcium-Dependent Activation of Soluble Adenylyl Cyclase in Parkin-Mutant Fibroblasts. Cells.

[42] Pacelli C., De Rasmo D., Signorile A., Grattagliano I., di Tullio G., D’Orazio A., Nico B., Comi G.P., Ronchi D., Ferranini E. (2011). Mitochondrial Defect and PGC-1α Dysfunction in Parkin-Associated Familial Parkinson’s Disease. Biochim. Biophys. Acta.

[43] Vergara D., Gaballo A., Signorile A., Ferretta A., Tanzarella P., Pacelli C., Di Paola M., Cocco T., Maffia M. (2017). Resveratrol Modulation of Protein Expression in Parkin-Mutant Human Skin Fibroblasts: A Proteomic Approach. Oxid. Med. Cell. Longev..

[44] Lippolis R., Siciliano R.A., Pacelli C., Ferretta A., Mazzeo M.F., Scacco S., Papa F., Gaballo A., Dell’Aquila C., De Mari M. (2015). Altered Protein Expression Pattern in Skin Fibroblasts from Parkin-Mutant Early-Onset Parkinson’s Disease Patients. Biochim. Biophys. Acta BBA Mol. Basis Dis..

[45] Lobasso S., Tanzarella P., Vergara D., Maffia M., Cocco T., Corcelli A. (2017). Lipid Profiling of Parkin-Mutant Human Skin Fibroblasts. J. Cell. Physiol..

[46] Guerra F., Girolimetti G., Beli R., Mitruccio M., Pacelli C., Ferretta A., Gasparre G., Cocco T., Bucci C. (2019). Synergistic Effect of Mitochondrial and Lysosomal Dysfunction in Parkinson’s Disease. Cells.

[47] Pacelli C., Rotundo G., Lecce L., Menga M., Bidollari E., Scrima R., Cela O., Piccoli C., Cocco T., Vescovi A.L. (2019). Parkin Mutation Affects Clock Gene-Dependent Energy Metabolism. Int. J. Mol. Sci..

[48] Signorile A., Ferretta A., Pacelli C., Capitanio N., Tanzarella P., Matrella M.L., Valletti A., De Rasmo D., Cocco T. (2021). Resveratrol Treatment in Human Parkin-Mutant Fibroblasts Modulates CAMP and Calcium Homeostasis Regulating the Expression of Mitochondria-Associated Membranes Resident Proteins. Biomolecules.

[49] Papalia T., Barreca D., Panuccio M.R. (2017). Assessment of Antioxidant and Cytoprotective Potential of Jatropha (*Jatropha curcas*) Grown in Southern Italy. Int. J. Mol. Sci..

[50] Muscolo A., Romeo F., Marra F., Mallamaci C. (2021). Recycling Agricultural, Municipal and Industrial Pollutant Wastes into Fertilizers for a Sustainable Healthy Food Production. J. Environ. Manag..

[51] Fossen T., Cabrita L., Andersen O.M. (1998). Colour and Stability of Pure Anthocyanins Influenced by PH Including the Alkaline Region. Food Chem..

[52] Muscolo A., Sidari M., Settineri G., Papalia T., Mallamaci C., Attinà E. (2019). Influence of Soil Properties on Bioactive Compounds and Antioxidant Capacity of Brassica Rupestris Raf. J. Soil Sci. Plant Nutr..

[53] De Rasmo D., Signorile A., Larizza M., Pacelli C., Cocco T., Papa S. (2012). Activation of the CAMP Cascade in Human Fibroblast Cultures Rescues the Activity of Oxidatively Damaged Complex I. Free Radic. Biol. Med..

[54] Signorile A., Santeramo A., Tamma G., Pellegrino T., D’Oria S., Lattanzio P., De Rasmo D. (2017). Mitochondrial CAMP Prevents Apoptosis Modulating Sirt3 Protein Level and OPA1 Processing in Cardiac Myoblast Cells. Biochim. Biophys. Acta Mol. Cell Res..

[55] Middleton E., Kandaswami C., Theoharides T.C. (2000). The Effects of Plant Flavonoids on Mammalian Cells: Implications for Inflammation, Heart Disease, and Cancer. Pharmacol. Rev..

[56] Wang W., Sun C., Mao L., Ma P., Liu F., Yang J., Gao Y. (2016). The Biological Activities, Chemical Stability, Metabolism and Delivery Systems of Quercetin: A Review. Trends Food Sci. Technol..

